# Conversion from Nonshockable to Shockable Rhythms and Out-of-Hospital Cardiac Arrest Outcomes by Initial Heart Rhythm and Rhythm Conversion Time

**DOI:** 10.1155/2020/3786408

**Published:** 2020-03-28

**Authors:** Wanwan Zhang, Shengyuan Luo, Daya Yang, Yongshu Zhang, Jinli Liao, Liwen Gu, Wankun Li, Zhihao Liu, Yan Xiong, Ahamed Idris

**Affiliations:** ^1^Department of Emergency Medicine, The First Affiliated Hospital of Sun Yat-sen University, Guangzhou, Guangdong, China; ^2^Department of Cardiology, The First Affiliated Hospital of Sun Yat-sen University, Guangzhou, Guangdong, China; ^3^Department of Hypertension and Vascular Disease, The First Affiliated Hospital of Sun Yat-sen University, Guangzhou, Guangdong, China; ^4^Department of Epidemiology, Johns Hopkins Bloomberg School of Public Health, Baltimore, MD, USA; ^5^Department of Emergency Medicine, University of Texas, Southwestern Medical Center, Dallas, TX, USA

## Abstract

**Background:**

The conversion from a nonshockable rhythm (asystole or pulseless electrical activity (PEA)) to a shockable rhythm (pulseless ventricular tachycardia or ventricular fibrillation) may be associated with better out-of-hospital cardiac arrest (OHCA) outcomes. There are insufficient data on the prognostic significance of such conversions by initial heart rhythm and different rhythm conversion time.

**Methods:**

Among 24,849 adult OHCA patients of presumed cardiac etiology with initial asystole or PEA in the Resuscitation Outcomes Consortium Cardiac Epidemiologic Registry (version 3, 2011–2015), we examined the association of shockable rhythm conversion with prehospital return of spontaneous circulation (ROSC), survival, and favorable functional outcome (modified Rankin Scale score ≤3) at hospital discharge by initial rhythm and rhythm conversion time (time from cardiopulmonary resuscitation (CPR) initiation by emergency medical providers to first shock delivery), using logistic regression adjusting for key clinical characteristics.

**Results:**

Of 16,516 patients with initial asystole and 8,333 patients with initial PEA, 16% and 20% underwent shockable rhythm conversions; the median rhythm conversion time was 12.0 (IQR: 6.7–18.7) and 13.2 (IQR: 7.0–20.5) min, respectively. No difference was found in odds of prehospital ROSC across rhythm conversion time, regardless of initial heart rhythm. Shockable rhythm conversion was associated with survival and favorable functional outcome at hospital discharge only when occurred during the first 15 min of CPR, for those with initial asystole, or the first 10 min of CPR, for those with initial PEA. The associations between shockable rhythm conversion and outcomes were stronger among those with initial asystole compared with those with initial PEA.

**Conclusions:**

The conversion from a nonshockable rhythm to a shockable rhythm was associated with better outcomes only when occurred early in initial nonshockable rhythm OHCA, and it has greater prognostic significance when the initial rhythm was asystole.

## 1. Introduction

The prognosis of out-of-hospital cardiac arrest (OHCA) remains poor [1–3]. OHCA patients with nonshockable rhythms (i.e., asystole and pulseless electrical activity (PEA)) are unlikely to benefit from an electrical defibrillation and suffer the worst outcomes [4]. Given that patients with nonshockable rhythms include the majority of presentations with the worst outcomes and represent the greatest opportunity to improve survival, the identification of prognostic factors in these patients is of clinical importance.

The conversion from a nonshockable rhythm to a shockable rhythm (i.e., pulseless ventricular tachycardia or ventricular fibrillation) has been shown to be associated with better short- or long-term outcomes in some, but not all OHCA populations [5–10]. In a previous meta-analysis involving 1,108,281 OHCA patients across 12 studies, we showed that the conversion from a nonshockable rhythm to a shockable rhythm and the subsequent electrical defibrillation attempt were associated with better outcomes only when the initial rhythm was asystole [5]. In a subgroup analysis, we found that the association between shockable rhythm conversion and 1-month favorable functional outcomes in patients with initial nonshockable rhythms tended to be weaker when rhythm conversion occurred late compared to early. In that analysis, however, only data from 2 studies, both conducted in Japan, were included [11, 12]. Findings were not stratified by initial heart rhythm, and the full spectrum of OHCA outcomes was not examined. Further investigation is thus needed to thoroughly assess the prognostic significance of shockable rhythm conversion by initial rhythm and rhythm conversion time and across different outcomes in initial nonshockable rhythm OHCA.

Using data from the Resuscitation Outcomes Consortium (ROC) Cardiac Epidemiologic Registry (version 3, 2011– 2015), a North American population-based registry that included more than 60,000 EMS-treated OHCA events from 264 Emergency Medical Service (EMS) agencies and per-protocol ascertainments of multiple outcomes, we sought to thoroughly investigate the associations of conversion from a nonshockable rhythm to a shockable rhythm and prehospital return of spontaneous circulation (ROSC), survival, and favorable functional outcome at hospital discharge, stratified by initial heart rhythm and across the spectrum of rhythm conversion time in OHCA patients with initial nonshockable rhythms.

## 2. Materials and Methods

### 2.1. Study Design and Population

**Figure 1:**
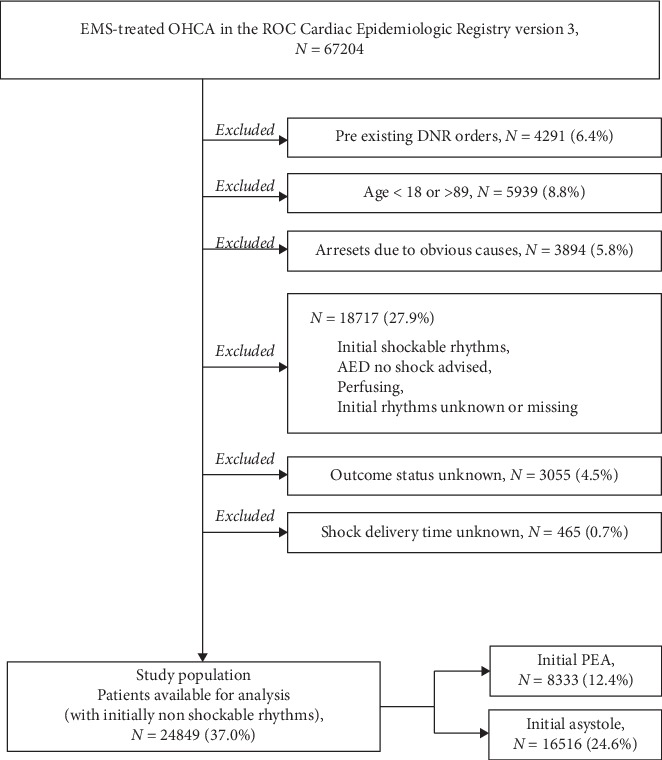
Study population selection process. EMS, Emergency Medical Services; OHCA, out-of-hospital cardiac arrest; ROC, Resuscitation Outcomes Consortium; DNR, do-not-resuscitate; AED, automatic external defibrillator; PEA, pulseless electrical activity; *N*, number.

This study is a secondary analysis using data of the Resuscitation Outcomes Consortium (ROC) Cardiac Epidemiologic Registry from April 2011 to June 2015 (version 3). ROC is a network of clinical research of out-of-hospital cardiac arrest consisting of ten North Regional Centers (Ottawa, Toronto, Vancouver, Birmingham, Dallas, Pittsburgh, Milwaukee, Portland, Seattle/King County, and San Diego) across the United States and Canada and their respective EMS systems [13–16]. The present study population was derived from 67,204 patients who were treated by EMS providers in the ROC Cardiac Epidemiologic Registry from April 2011 to June 2015 (version 3). Patients with the following characteristics were included in the present study: age between 18 and 89 years, no existing do-not-resuscitate order, cardiac arrest of no obvious causes (presumed cardiac etiology), known initial rhythm, shock delivery status, and documented OHCA outcomes (Figure 1).

Study data were obtained from the National Institutes of Health (NIH) Biological Specimen and Data Repository Information Coordinating Center (https://biolincc.nhlbi.nih.gov/studies/roc_cardiac_epistry_3/?q=roc). The present study is a retrospective, observational analysis of this dataset approved by the Institutional Review Boards (IRBs) of ROC and NIH and then downloaded from the NIH website. The requirement of written informed consent was waived because of the nature of an anonymous dataset.

## 3. Methods of Measurements

The first recorded electrical defibrillation delivery during cardiopulmonary resuscitation (CPR) was used as the surrogate for the conversion from a nonshockable rhythm to a shockable rhythm. The time of shockable rhythm conversion was defined as the interval from the first chest compression by an EMS provider to the time of the first electrical shock delivery. Time-stamped data (hours: minutes: seconds) on chest compression initiation and shock deliveries were automatically recorded by monitor-defibrillators. All other covariables were ascertained based on standard ROC Cardiac Epidemiologic Registry protocols.

### 3.1. Outcomes

Three outcomes were assessed in this study: prehospital ROSC, survival to hospital discharge, and favorable functional outcome at hospital discharge, which was defined as a modified Rankin Scale score of ≤3. Outcomes were ascertained by research personnel at each participating center through review of prehospital data streams, audio recordings, and hospital records. Modified Rankin Scale scores at hospital discharge were assigned using a standardized chart review instrument.

### 3.2. Data Analysis

Patient characteristics overall and stratified by initial rhythm (asystole or PEA) and categories of rhythm conversion time were summarized using descriptive statistics. The associations between shockable rhythm conversion (compared to no rhythm conversion) and outcomes were assessed using logistic regression with adjustment for age, sex, witness status (not witnessed vs. bystander witnessed vs. witnessed by EMS personnel), bystander CPR, location of OHCA (public vs. nonpublic), use of advanced airway, EMS response time, and use and dosage of epinephrine. Shockable rhythm conversion time was first modelled as a continuous variable, and cubic splines with knots at the 5^th^, 35^th^, 65^th^, and 95^th^ percentiles and the referent point at the 35^th^ percentile (conversion time = 10 min), were used to visualize associations across conversion time. Rhythm conversion time was then categorized (<10 min, 10–15 min, and ≥15 min), and logistic regressions were repeated, comparing shockable rhythm conversion with nonshockable rhythm conversion, by conversion time categories. All analyses by continuous or categorical rhythm conversion time were stratified by initial rhythm (asystole or PEA). A two-sided *α*-value of 0.05 was chosen as the cutoff for statistical significance. Statistical analyses were conducted using SPSS 20.0 (IBM Inc., Armonk, New York) and Stata 15.1 (StataCorp, College Station, Texas).

## 4. Results

### 4.1. Patient Characteristics

Of the 24,849 patients with initial nonshockable rhythm OHCA, 16,516 (66%) had initial asystole and 8,333 (34%) had initial PEA (Table 1). Among patients with initial asystole and those with initial PEA, respectively, the median age was 66 (IQR: 54–77) and 70 (IQR: 59–79), 10,224 (62%) and 5,120 (61%) were men, 2,581 (16%) and 1,655 (20%) underwent shockable rhythm conversions, and the median rhythm conversion time was 12.0 (IQR: 6.7–18.7) and 13.2 (IQR: 7.0–20.5) min. Patient characteristics were comparable between those with initial asystole and those with initial PEA, except that the proportion of patients with OHCA witnessed by EMS personnel or bystander was higher among those with initial PEA. There was no statistical difference in patient characteristics across rhythm conversion time among those who underwent shockable rhythm conversions.

### 4.2. Shockable Rhythm Conversion and Prehospital ROSC

**Figure 2:**
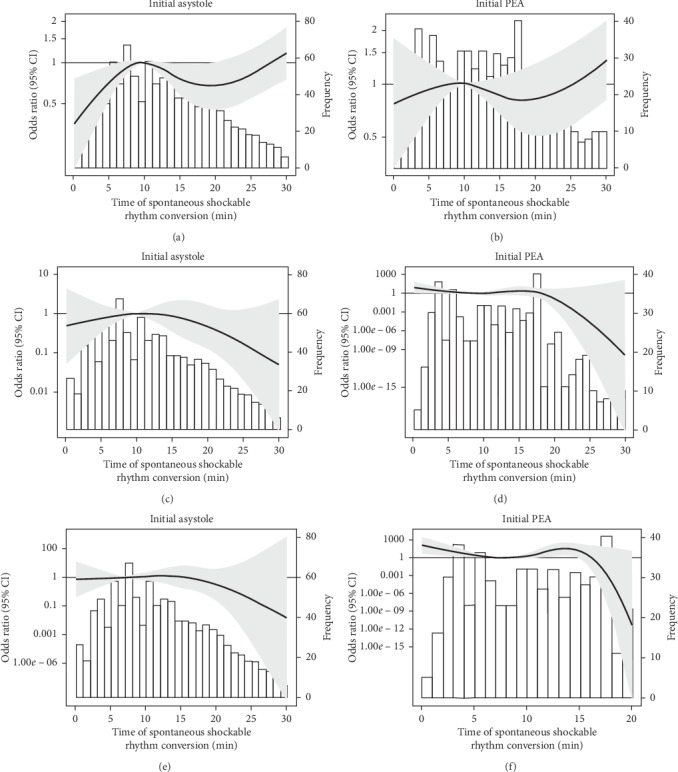
Adjusted odds ratios and 95% confidence intervals comparing shockable rhythm conversion and nonshockable rhythm conversion for prehospital return of spontaneous circulation, survival to hospital discharge, and favorable functional outcome at hospital discharge by time of rhythm conversion in initial heart rhythm in the ROC Cardiac Epidemiologic Registry (version 3). (a) Adjusted odds ratios and 95% confidence intervals for prehospital return of spontaneous circulation in OHCA patients with initial asystole or PEA. (b) Adjusted odds ratios and 95% confidence intervals for survival to hospital discharge in OHCA patients with initial asystole or PEA. (c) Adjusted odds ratios and 95% confidence intervals for favorable functional outcome at hospital discharge in OHCA patients with initial asystole or PEA. OHCA, out-of-hospital cardiac arrest; PEA, pulseless electrical activity; CI, confidence interval.

Among patients with initial asystole (*N* = 16,516) and those with initial PEA (*N* = 8,333), respectively, 3,361 (20%) and 3,106 (37%) had prehospital ROSC. Of the 1061, 567, and 953 patients with shockable rhythm conversions from initial asystole at <10 min, 10–15 min, and ≥15 min of CPR, 323 (30%), 164 (29%), and 322 (34%), respectively, underwent prehospital ROSC. Of the 616, 330, and 705 patients with shockable rhythm conversions from initial PEA at <10 min, 10–15 min, and ≥15 min of CPR, 238 (39%), 123 (37%), and 278 (39%), respectively, underwent prehospital ROSC (Table 2). Using the 35^th^ percentile of rhythm conversion times (10 min) as the referent point, there was a trend towards increasing odds of prehospital ROSC with rhythm conversion time, when the initial rhythm was asystole and shockable rhythm conversion occurred within 10 min (Figure 2(a)), after adjustment for age, sex, witness status, bystander CPR, OHCA location, use of advanced airway, EMS response time, and use of epinephrine. After categorizing rhythm conversion time into <10 min, 10–15 min, and ≥15 min of CPR and using nonshockable rhythm conversion as the reference, however, there was no observable difference in association between shockable rhythm conversion and prehospital ROSC by rhythm conversion time among those with asystole (Table 2). Among those with initial PEA, the association between shockable rhythm conversion and prehospital ROSC was weaker compared to those with initial asystole and did not differ by rhythm conversion time.

### 4.3. Shockable Rhythm Conversion and Survival to Hospital Discharge

Among patients with initial asystole (*N* = 16,516) and those with initial PEA (*N* = 8,333), respectively, 173 (1%) and 295 (4%) survived to hospital discharge. Of the 1061, 567, and 953 patients with shockable rhythm conversions from initial asystole at <10 min, 10–15 min, and ≥15 min of CPR, 34 (3%), 12 (2%), and 9 (1%), respectively, survived to hospital discharge. Of the 616, 330, and 705 patients with shockable rhythm conversions from initial PEA at <10 min, 10–15 min, and ≥15 min of CPR, 34 (6%), 12 (4%), and 11 (2%), respectively, survived to hospital discharge. Using the 35^th^ percentile of rhythm conversion times (10 min) as the referent point, there was a linear trend towards decreasing odds of survival to hospital discharge with rhythm conversion time for both patients with initial asystole and those with initial PEA, adjusting for covariates (Figure 2(b)). After categorizing rhythm conversion time into <10 min, 10–15 min, and ≥15 min of CPR, higher odds of survival to hospital discharge was observed with shockable rhythm conversion, when the initial rhythm was asystole and shockable rhythm conversion occurred within the first 10 min (odds ratio (OR) 4.39; 95% confidence interval (CI): 2.95, 6.53) or 10–15 min of CPR (OR 3.05; 95% CI: 1.65, 5.62), or when the initial rhythm was PEA and shockable rhythm conversion occurred within the first 10 min of CPR (OR 2.09; 95% CI: 1.42, 3.08).

### 4.4. Shockable Rhythm Conversion and Favorable Functional Outcome

Among patients with initial asystole (*N* = 16,516) and those with initial PEA (*N* = 8,333), respectively, 70 (0.4%) and 153 (2%) had a favorable functional outcome at hospital discharge. Of the 1061, 567, and 953 patients with shockable conversions from initial asystole at <10 min, 10–15 min, and ≥15 min of CPR, 14 (1%), 7 (1%), and 2 (0.2%), respectively, had a favorable functional outcome at discharge. Of the 616, 330, and 705 patients with shockable conversions from initial PEA at <10 min, 10–15 min, and ≥15 min of CPR, 20 (3%), 3 (1%), and 4 (1%), respectively, had a favorable functional outcome at discharge. Using the 35^th^ percentile of rhythm conversion times (10 min) as the referent point, there was a trend towards decreasing odds of favorable functional outcome at discharge with shockable rhythm conversion time, most prominently when occurred beyond the first 15 min of CPR among those with initial asystole, adjusting for all covariates (Figure 2(c)). After categorizing rhythm conversion time into <10 min, 10–15 min, and ≥15 min of CPR, higher odds of favorable functional outcome at discharge was observed with shockable rhythm conversion, when the initial rhythm was asystole and conversion occurred within the first 10 min (OR 4.28; 95% CI: 2.32, 7.89) or 10–15 min of CPR (OR 4.38; 95% CI: 1.94, 9.90), or when the initial rhythm was PEA and conversion occurred within the first 10 min (OR 2.26; 95% CI: 1.37, 3.75).

## 5. Discussion

In this retrospective analysis of 24,849 OHCA patients with initial nonshockable rhythms in a North American population-based registry, we found that shockable rhythm conversion was associated with survival and better functional outcomes at hospital discharge in patients with initial asystole, only when rhythm conversion occurred within the first 15 min of CPR. In patients with initial PEA, shockable rhythm conversion was associated with survival and better functional outcomes at hospital discharge, only when occurred within the first 10 min of CPR, and the associations were weaker compared to among those with initial asystole.

The conversion from a nonshockable rhythm to a shockable rhythm in OHCA remains a subject of clinical importance. Some studies have demonstrated strong associations between shockable rhythm conversion and better outcomes in OHCA patients with initial nonshockable rhythms, whereas others did not [6–10]. Factors underlying the differing prognostic significance of shockable rhythm conversion across populations have been relatively understudied, and there has been little published data on the interactions across initial heart rhythm, rhythm conversion time, and shockable rhythm conversion in initial nonshockable rhythm OHCA. To our knowledge, only two studies have thus far analyzed data on shockable rhythm conversion and outcomes stratified by rhythm conversion time. Goto et al. studied 569,937 OHCA patients enrolled in a Japanese national registry between 2005 and 2010 [12], and Funada et al. studied 430,443 OHCA patients enrolled in the same registry between 2011 and 2014 [11]. Both studies involved only Japanese patients, categorized rhythm conversion times into 10-min intervals, assessed outcomes at one-month post-OHCA and did not stratify analyses by initial arrest rhythm (which has previously been shown to interact with shockable rhythm conversion for its associations with OHCA outcomes) [5, 17]. These researchers concluded that the first 20 min of CPR could be a threshold beyond which shockable rhythm conversion may no longer be associated with better outcomes in OHCA patients with initial nonshockable rhythms [11, 12]. In contrast to these studies, the present study provides a more thorough delineation of the prognostic significance of shockable rhythm conversion stratified by initial heart rhythm, across the continuous spectrum of rhythm conversion time, and multiple OHCA outcomes that were assessed from at the field till hospital discharge.

Our findings may have clinical implications and provide a basis for the development of better CPR strategies. The current *American Heart Association Guidelines for Cardiopulmonary Resuscitation and Emergency Cardiovascular Care* recommend “appropriate rhythm-based strategies” for patients whose heart rhythms have evolved during CPR [18], which would indicate attempts to electrical defibrillation in patients who had undergone shockable rhythm conversions from nonshockable rhythms. However, as demonstrated in the present study, when such rhythm conversions occurred beyond certain time thresholds (i.e., 15 min for initial asystole and 10 min for initial PEA), electrical shocks may no longer confer survival or functional outcome benefits, possibly because the arresting heart had entered a “metabolic phase” where there was irreversible ischemic damage, and the heart muscles had become more susceptible to reperfusion injury [19]. Continued chest compressions to maximize circulation, in these scenarios, may therefore be preferable to electrical defibrillation attempts.

Strengths of this study include its large sample size and per-protocol ascertainment of shock delivery time and multiple OHCA outcomes. However, our study has several limitations. First, because our data originated from a North American registry, the generalizability of our findings to other populations may be limited. Second, like all observational studies, our findings may be affected by uncontrolled confounding. Nonetheless, because of the rigorous design of the ROC Cardiac Registry Epistry and its focus on per-protocol ascertainment of pertinent OHCA covariables and outcomes, we believe the influence of confounding and measurement errors was reduced to the greatest extent possible. Third, only data collected after the initiation of CPR by EMS personnel were available. We are thus unable to account for the duration of cardiac arrest or CPR performed before EMS arrival. Fourth, we did not have access to continuous heart rhythm readings and the use of first electrical shock delivery as the surrogate for shockable rhythm conversion may result in misclassifications. Further, without heart rhythm readings, we were unable to ascertain whether shockable rhythm conversions resulted in fine ventricular fibrillations, as opposed to coarse ventricular fibrillations, which are more likely to respond to electrical shocks. Nonetheless, all EMS providers participating in the ROC were instructed to adhere to clinical practice guidelines, minimizing the chances of inappropriate delivery of shocks in the absence of shockable heart rhythms.

In conclusion, the conversion from a nonshockable rhythm to a shockable rhythm was associated with better outcomes only when occurred early in initial nonshockable rhythm OHCA. These findings may facilitate the advancement of OHCA resuscitation strategies.

## Figures and Tables

**Table 1:** OHCA patient characteristics overall, by initial rhythm, and by time of spontaneous shockable rhythm conversion.

Variable	Overall	Initial asystole					Initial pulseless electrical activity				
		No rhythm conversion	Spontaneous rhythm conversion				No rhythm conversion	Spontaneous rhythm conversion			
			Total	Conversion in <10 min	Conversion in 10–15 min	Conversion in ≥15 min		Total	Conversion in <10 min	Conversion in 10–15 min	Conversion in ≥15 min
*N*	24849	13935	2581	1061	567	953	6678	1655	616	330	705
Median age, year (IQR)	67 (55, 78)	66 (54, 77)	65 (54, 76)	65 (55, 77)	64 (55, 76)	65 (53, 76)	70 (59, 79)	70 (58, 79)	69 (56, 79)	69 (59, 79)	71 (60, 79)
Men, *n* (%)	15344 (61.7)	8498 (61.0)	1726 (66.9)	736 (69.4)	383 (67.5)	607 (63.7)	4026 (60.3)	1094 (66.1)	435 (70.6)	222 (67.3)	437 (61.6)
Witnessed OHCA											
By EMS, *n* (%)	2492 (10.0)	601 (4.3)	114 (4.4)	46 (4.3)	24 (4.2)	44 (4.6)	1479 (22.1)	298 (18.0)	112 (18.2)	61 (18.5)	125 (17.6)
By bystander, *n* (%)	7747 (31.2)	3260 (23.4)	889 (34.4)	374 (35.2)	200 (35.3)	315 (33.1)	2833 (42.4)	765 (46.2)	275 (44.6)	158 (47.9)	332 (46.8)
Bystander resuscitation, *n* (%)	10738 (43.2)	6280 (45.1)	1147 (44.4)	481 (45.3)	254 (44.8)	412 (43.2)	2612 (39.1)	699 (42.2)	286 (46.4)	139 (42.1)	274 (38.6)
Public location, *n* (%)	2426(9.8)	1060 (7.6)	301 (11.7)	129 (12.2)	73 (12.9)	99 (10.4)	802 (12.0)	263 (15.9)	125 (20.3)	48 (14.5)	90 (12.7)
Median EMS response time, min (IQR)	5.4 (4.2, 6.9)	5.3 (4.1, 6.8)	5.5 (4.2, 7.0)	5.5(4.3, 7.0)	5.4 (4.1, 7.0)	5.4 (4.1, 7.0)	5.5 (4.2, 7.0)	5.6 (4.3,7.0)	5.6 (4.3, 7.0)	5.5 (4.2, 7.0)	5.7 (4.3, 7.0)
Median epinephrine use, mg (IQR)	3 (3, 5)	3(3, 4)	4(3, 6)	4(3, 5)	4 (3, 5)	4 (3, 6)	3 (2, 4)	4(3, 6)	4 (3, 6)	4 (3, 5)	5(3, 6)
Advanced airway applied, *n* (%)	21592 (86.9)	11964 (85.9)	2341 (90.7)	933 (87.9)	512 (90.3)	896 (94.0)	5807 (87.0)	1480 (89.4)	530 (86.0)	289 (87.6)	661 (93.2)
Median time of rhythm conversion, min (IQR)	—	—	12.0 (6.7, 18.7)	6.0 (3.9, 7.9)	12.4 (11.2, 13.7)	21.4 (17.9, 26.5)	—	13.2 (7.0, 20.5)	5.5 (3.1, 7.7)	12.6 (11.1, 14.0)	21.9 (18.4, 26.8)
Median time of rhythm conversion, min (IQR)	—	—	12.0 (6.7, 18.7)	6.0 (3.9, 7.9)	12.4 (11.2, 13.7)	21.4 (17.9, 26.5)	—	13.2 (7.0, 20.5)	5.5 (3.1, 7.7)	12.6 (11.1, 14.0)	21.9 (18.4, 26.8)

OHCA, out-of-hospital cardiac arrest; EMS, Emergency Medical Services; *n*, number; N, total number; IQR, interquartile range; min, minute; mg, milligram.

**Table 2:** Results from multivariable logistic regression analysis, assessing the associations of spontaneous rhythm conversion with prehospital ROSC, survival to hospital discharge, and favorable functional outcome in initial nonshockable rhythm OHCA stratifying by time of spontaneous shockable rhythm conversion.

	*N* total	Prehospital ROSC		Survival to hospital discharge		Favorable functional outcome at hospital discharge	
		*N* of events (proportion, %)	OR (95% CI)	*N* of events (proportion, %)	OR (95% CI)	*N* of events (proportion, %)	OR (95% CI)
Initial asystole							
No spontaneous rhythm conversion	13935	2552 (18.2)	Reference	118 (0.8)	Reference	47 (0.3)	Reference
Spontaneous conversion in <10 min	1061	323 (30.4)	1.93 (1.67, 2.23)	34 (3.2)	4.39 (2.95, 6.53)	14 (1.3)	4.28 (2.32, 7.89)
Spontaneous conversion in 10–15 min	567	164 (28.9)	1.76 (1.45, 2.13)	12 (2.1)	3.05 (1.65, 5.62)	7 (1.2)	4.38 (1.94, 9.90)
Spontaneous conversion in ≥15 min	953	322 (33.8)	2.23 (1.92, 2.59)	9 (0.9)	1.60 (0.80, 3.20)	2 (0.2)	0.90 (0.22, 3.74)

Initial pulseless electrical activity							
No spontaneous rhythm conversion	6678	2467 (36.9)	Reference	238 (3.6)	Reference	126 (1.9)	Reference
Spontaneous conversion in <10 min	616	238 (38.6)	1.26 (1.06, 1.50)	34 (5.5)	2.09 (1.42, 3.08)	20 (3.2)	2.26 (1.37, 3.75)
Spontaneous conversion in 10–15 min	330	123 (37.3)	1.15 (0.91, 1.45)	12 (3.6)	1.50 (0.82, 2.77)	3 (0.9)	0.72 (0.23, 2.33)
Spontaneous conversion in ≥15 min	705	278 (39.2)	1.32 (1.12, 1.56)	11 (1.6)	0.88 (0.47, 1.65)	4 (0.6)	0.67 (0.24, 1.85)

Covariables in regression models include age, sex, witnessed OHCA (by EMS vs. bystander vs. not), bystander CPR, location of OHCA (public vs. not), use of advanced airway, Emergency Medical Services response time, and dose of epinephrine administered. Favorable functional outcome at hospital discharge is defined as a Modified Rankin Scale score of ≤3. OHCA, out-of-hospital cardiac arrest; EMS, Emergency Medical Services; CPR, cardiopulmonary resuscitation; N, number; min, minute; OR, odds ratio.

## Data Availability

The data that support the findings of this study are openly available in the National Institutes of Health (NIH) Biological Specimen and Data Repository Information Coordinating Center (https://biolincc.nhlbi.nih.gov/studies/roc_cardiac_epistry_3/). Requests for the date can be submitted through the website of the National Institutes of Health (NIH): https://biolincc.nhlbi.nih.gov/studies/roc_cardiac_epistry_3/ (https://biolincc.nhlbi.nih.gov/studies/rocprimed/?q=primed).
